# Nature of the low magnetization decay on stacks of second generation superconducting tapes under crossed and rotating magnetic field experiments

**DOI:** 10.1038/s41598-018-19681-8

**Published:** 2018-01-22

**Authors:** Mehdi Baghdadi, Harold S. Ruiz, Timothy A. Coombs

**Affiliations:** 1 University of Greenwich, Faculty of Engineering and Science, London, SE10 9LS United Kingdom; 2 University of Cambridge, Department of Engineering, Cambridge, CB3 0FA United Kingdom; 3 University of Leicester, Department of Engineering, Leicester, LE1 7RH United Kingdom

## Abstract

The extremely low decay factor on the trapped magnetic field by stacks of second-generation high-temperature superconducting tapes reported in Appl. Phys. Lett. 104, 232602 (2014), is in apparent contradiction with the classical results for the demagnetization of superconducting bulks and thin films, where the samples undergo a severe and progressive decay under crossed magnetic field conditions. Nevertheless, in this paper, we demonstrate how the theoretical approaches and experimental measurements on superconducting bulks, thin films, and stacks of superconducting tapes can be reconciled, not only under the crossed field configuration but also under rotating magnetic field conditions, by showing that the stacks of commercial tapes behave as a system of electrically unconnected layers preventing the deformation of profiles of current along its external contour. This study extends up to the consideration of using novel superconducting/ferromagnetic metastructures, where soft ferromagnetic films are interlayered, reporting a further reduction on the magnetization decay of about 50% in the crossed field configuration. Remarkably, after applying the same number of cycles either of rotating or crossed magnetic field to these metastructures, the difference between the magnetization decay is found to be negligible, what demonstrates their highly superior performance when compared to conventional stacks of superconducting tapes.

## Introduction

Recent experiments on stacks of second generation high temperature superconducting (2G-HTS) tapes have revealed their viability for the replacement of high-field permanent magnets and superconducting (SC) bulks, in a wide variety of engineering applications^1–5^. Currently, an outstandingly high magnetic field of 17.6 T at 26 K has been trapped in a mechanically reinforced stack of two silver-doped GdBCO SC-bulks, each 25 mm in diameter, and 15 mm high, breaking a world record which had stood for ten years^6^. On the other hand, a double 12 mm square stack of commercial 2G-HTS tapes, each 6.9 mm height, has been shown to be capable of trapping up to 7.34 T at 4.2 K (~5.7 T at 26 K), opening up the possibility of attaining trapped magnetic fields greater that the current world record for SC bulks, if 40 mm-wide commercial tapes were used^5^. Moreover, unlike the most recent observations on stacks of 2G-HTS tapes^4^, it has been already proven, both experimentally and theoretically, that the intensity of the trapped magnetic field in SC bulks can be strongly reduced by the so-called collapse of the magnetization phenomenon, either in the crossed- or rotating- magnetic field configuration^7–15^ i.e, when the SC sample is exposed to an oscillating time-varying magnetic flux oriented in orthonormal direction to the original magnetization.

Numerical approaches in the past have indicated that very thin SC films are also prone to decay in trapped magnetic field after a few cycles of a crossed field of low intensity^16^. However, we have recently demonstrated that a strong reduction in the decay factor (≳95%) can be achieved when using a stack of sixteen 12 mm square 2G-HTS tapes. This is true even after 100 cycles of a transverse crossed magnetic field, 2.5 times greater than the maximum trapped field^4^. This finding, is remarkable because under the same experimental conditions (overall sample dimensions and applied magnetic field), the original magnetization of a SC bulk can be seen to have been reduced by more than 50% after just one cycle of the external magnetic excitation^7^. However, our finding has also highlighted the fact that there is a clear breakpoint between the well-known electromagnetic theory for the phenomenon of collapse of the magnetization in SC bulks, and its counterpart for stacks of SC tapes. Thus, in order to reassemble the physical understanding of the demagnetization phenomena in SC bulks and stacks of SC tapes, in this paper we present the theoretical explanation of the experimental observations for the crossed-field experiments reported elsewhere^4^, and for the sake of generality, our study has been extended for considering also scenarios with rotating magnetic field configurations. Then, after demonstrating, theoretically and experimentally, the advantages of using stacks of SC tapes concerning to the demagnetization processes, we introduce a novel metastructure composed of a stack of SC films interlayered with soft-ferromagnetic layers (FM) which leads to even better performance under the crossed and rotating magnetic field configurations.

### Theoretical framework

**Figure 1:**
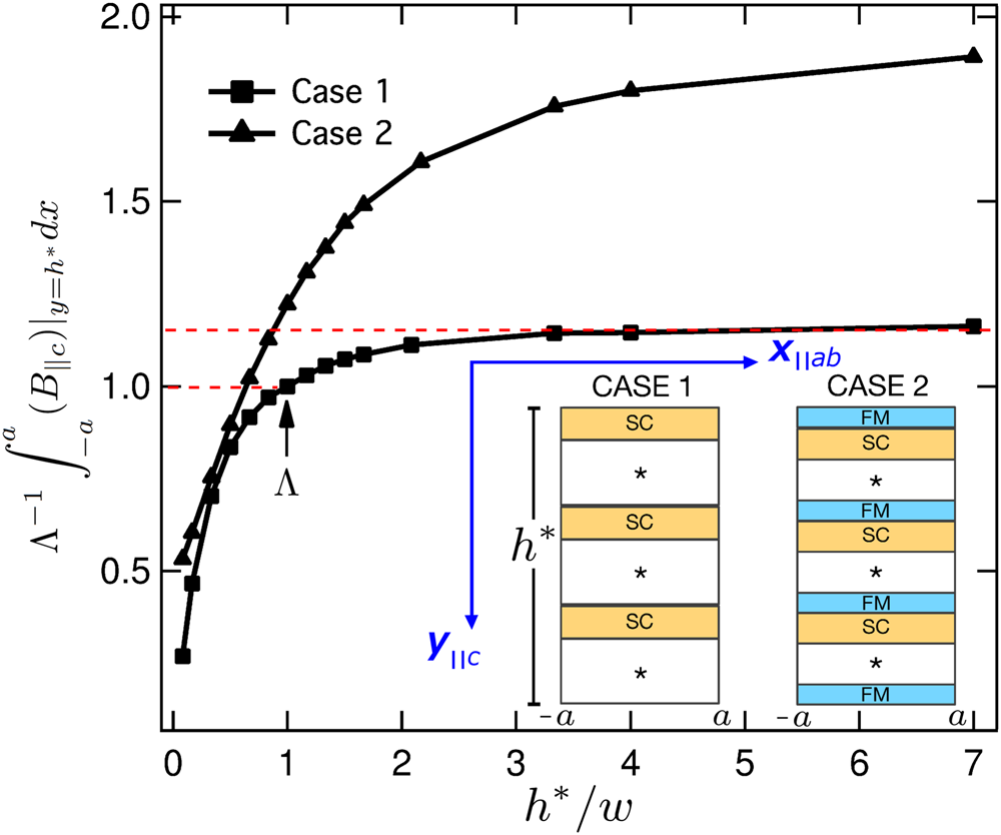
Remanent magnetic flux integrated over the uppermost surface of a fully premagnetized stack of tapes in the configurations illustrated at the inset. All results have been renormalized in terms of the integrated value, ${\rm{\Lambda }}=8.665$ T.mm, it calculated from Case-1, when h*/*w* = 1.

For the numerical solution of the Maxwell equations, in this paper we present a 2D theoretical model based on the conventional framework of the **H**-Formulation^12,14^. In order to isolate the nature of the discrepancies between the demagnetization processes in SC bulks and stacks of SC tapes, we initially consider that the stack of 2G-HTS tapes has been fully premagnetized along the *c-axis*, i.e., perpendicular to the widest surface of the tapes (Fig. 1) and then, two different configurations for the transverse external magnetic field (crossed and rotating field experiments) are applied.

The theoretical framework of the *H*-formulation has been published elsewhere^12,14^, but the conditions presented here to solve the current problem are not constrained to this particular formulation. In detail, we have considered a stack of *N* superconducting tapes, each 1 *μ*m thickness, and 12 mm width, with an interlayer separation of 55 *μ*m in order to consider the space allocated by the remaining (*) layers (cap layers, buffer stack, and substrate) composing a conventional stabilizer free 2G-HTS tape^17^ (Fig. 1, Case 1). The stack of SC tapes is translational invariant along the z-direction, i.e., with its length perpendicularly oriented to the direction of the applied magnetic field *H*_*a*_. Therefore, the induced current density J = *J*(*x*, *y*)*z* generates a planar magnetic field *H* which has no *z* component, and consequently, magnetic relaxation effects under the framework of the flux cutting mechanism do not need to be considered^10^. Additionally, we have considered a second case (Fig. 1, Case 2) on which soft-ferromagnetic interlayers (*μ*_*r*_ = 100), each 20 *μ*m thick, are added to the conventional stack of SC tapes (Case 1), it in order to prevent further deformations of the trapped magnetic flux after considering the crossed or rotating magnetic field experiments.

For the SC specimens we have assumed that the local flux density *B* is larger than the lower critical field B_*c*1_ of the superconductor, allowing us to use the expression **B** = *μ*_0_**H** over the entire space of elements. Also, for describing the typical flux-creep behaviour of the SC material, the following E-J power law,

1 $${\bf{E}}({\bf{J}},{\bf{B}})={E}_{0}\frac{{\bf{J}}}{|{\bf{J}}|}{(\frac{|{\bf{J}}|}{{J}_{c}(|{\bf{B}}|)})}^{n}\mathrm{\ ,}$$

under the standard electric field criterion *E*_0_ = 1 *μ*V/cm, with *n* = 21 for melt-textured YBCO at 77 K^14^, and the Kim’s model, ${J}_{c}(|{\bf{B}}|)={J}_{c}^{\ast }\mathrm{/(1}+|{\bf{B}}|/{B}^{\ast })$, has been considered. It is to be noticed, that the parameters ${J}_{c}^{\ast }$ and B* are by definition empirical parameters depending on the physical microstructure of the material, and are only considered here in order to get a more realistic estimation of the actual critical current density at a local level. On the other hand, to overcome the intrinsic difficulties associated with the high aspect ratio of the SC tape, (*h*/*w*)^−1^ = 12000, and to diminish the computing time, the thickness of each tape has been rescaled by a factor of 10 and therefore, the threshold value for the critical current density (${J}_{c}^{\ast }=2.3$ MA/cm^2^) has been rescaled accordingly.

Firstly, we have analysed the premagnetization stage of the stack of SC tapes as a function of the overall aspect ratio h^*^/*w*, for the two cases illustrated in Fig. 1. For case 1, it can be seen that the total magnetic flux (integrated over the uppermost surface of the stack of tapes) tends to saturate once the stack height is greater than 2, and rapidly decays if h^*^/*w* < 1. Therefore, we have determined that the optimal band for sizing the height of a stack of 2 G HTS tapes is $1\le {h}^{\ast }/w\le 2$. Strikingly, keeping about the same overall aspect-ratio and number of SC tapes before (Case 1) and after the inclusion of the soft-ferromagnetic layers (Case 2), we have found that under the same operative conditions, the SC/FM metastructure can trap a greater magnetic field with an increased performance of about 22% when h^*^/*w* = 1, and even greater than 50% when h^*^/*w* = 4, saturating at about ${h}^{\ast }/w\ge 8$. This is an interesting result, as it paves a new route for the manufacturing of much more efficient structures aiming for the trapping of strong magnetic fields in the near future.

**Figure 2:**
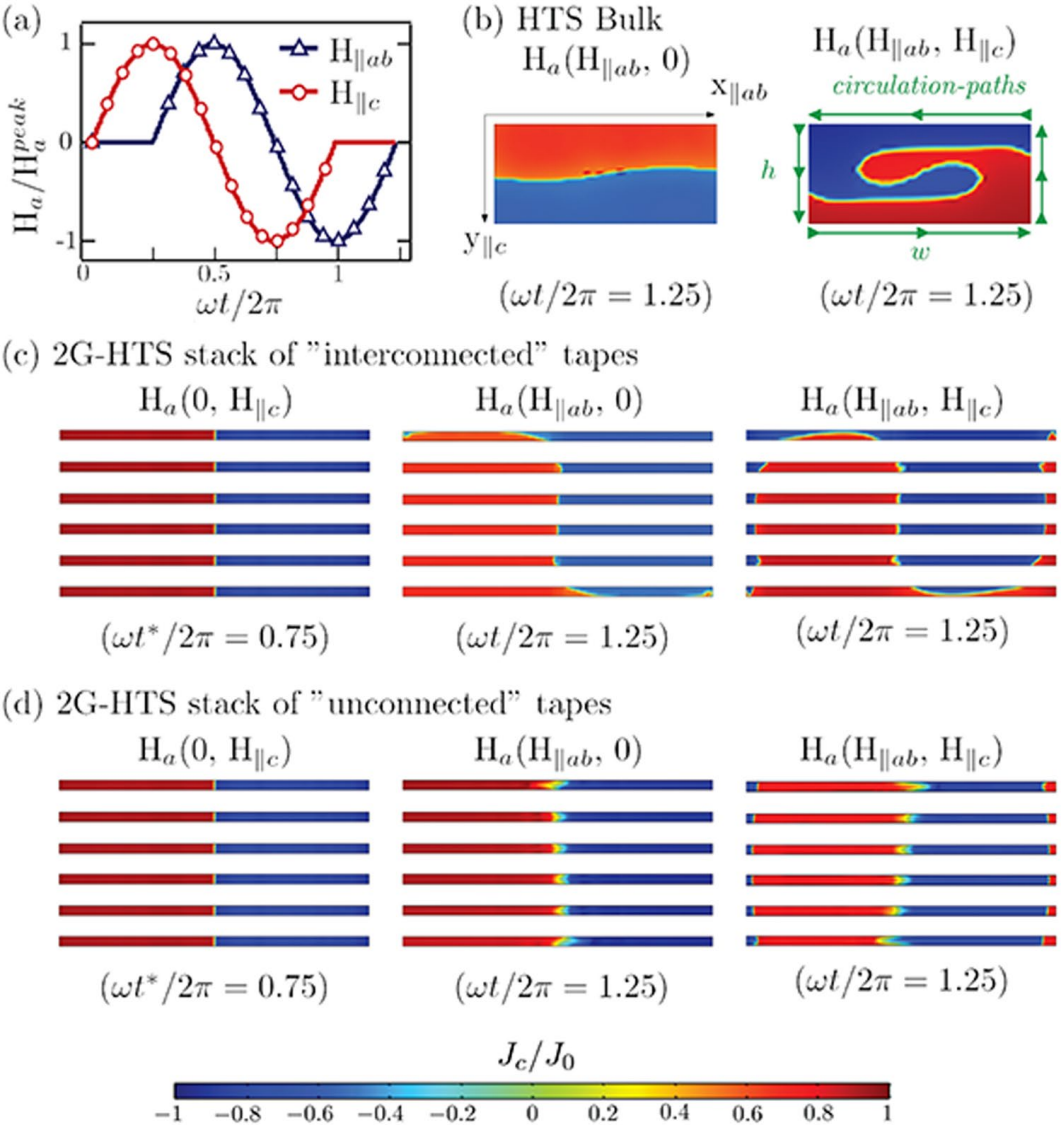
(**a**) Time dependence for the applied magnetic field in the crossed field configuration, ${H}_{a}={H}_{\parallel ab}$, and the rotating field configuration, ${H}_{a}={H}_{\parallel ab}+{H}_{\parallel c}$, with peak field values of ${\mu }_{0}{H}_{a}^{peak}=300$ mT. (**b**) Distribution of profiles of current inside of a SC bulk of aspect ratio ${(h/w)}^{-1}=3$ (not to scale), and ${J}_{c}^{\ast }=7$ kA/cm^2^, after applying one cycle of *H*_a_ under the crossed and rotating field configurations, respectively. Analogously, for the case of a stack of six 2G-HTS tapes, each with ${J}_{c}^{\ast }=2.3$ MA/cm^2^, the distribution of profiles of current (not to scale) is shown for the (**c**) *interconnected* and (**d**) *unconnected* ansatz, both starting from the same premagnetization condition depicted at the left of each row, respectively. All samples have been premagnetized such that the peak of trapped magnetic field before the cross and rotating field experiments is of 120 mT with a full penetration of the magnetic field, in good agreement with reported experimental measurements^4^.

Secondly, after achieving the full magnetization of the stack of SC tapes, we continue our study by considering the application of an external magnetic field transverse to the direction of the magnetization currents, in the crossed and rotating field configurations (Fig. 2). Likewise, for the sake of discussion, a SC-bulk capable to trap the same amount of magnetic field than in the case of a stack of 2G-HTS tapes has been considered. Both systems are initially characterized by the same kind of pattern for the profiles of current across the overall cross-section of the sample, i.e., with the flux front profile, *J*_*z*_ = 0, defined as a centered axisymmetric straight line along the y-axis. The main quantitative difference here, refers to the self-field critical current density of the SC bulk (${J}_{c}^{\ast }=\mathrm{7\ }kA/cm$^2^), which is much lesser than the one measured for a 2G-HTS tape^14,15^. Then, for the crossed-field configuration a series of transverse magnetic field cycles parallel to the *x*–axis were applied, and the two resultant magnetic field components (*H*_*x*_, *H*_*y*_) were computed. Analogously, the time-path for the applied magnetic field in the rotating field configuration is shown in Fig. 2(a), and the resulting distribution of profiles of current for both cases is displayed.

Essentially, under the framework of the H-Formulation^12,14^, the standard boundary conditions for solving the partial differential equation system (PDES) defined by Ampère and Faraday’s laws, together with the material laws remain unchanged. Therefore, the collapse of the magnetization for SC bulks under the crossed and rotating field configurations is straightforwardly achieved (Fig. 2(b)). This is already a classical result^7,8,11–16^, which intriguingly does not seem to agree with the experimental evidence for stacked 2G-HTS tapes^4^, in a first instance. Nevertheless, having in mind that the overall sum of profiles of current (*J*_*i*_) inside of a SC specimen of cross-section area Ω, is by definition equal to zero (in the absence of a transport current), two different scenarios can be envisaged for the stack of SC tapes, with only one being physically correct. On the one hand, coupling currents of the same order of magnitude than *J*_c_ may appear through a conductive matrix^7,18–21^, then, the SC tapes or the filaments they are composed from behave as electrically interconnected elements satisfying the condition $\int {J}_{i}d{\rm{\Omega }}=0$, where Ω stands for the overall cross-section area of the stack. Thus, under this ansatz, we have obtained that the stack of tapes acts as a single SC-bulk of relative dimensions h*/*w* (Fig. 2(c)), allowing us to explain, simultaneously, the recent results reported in other studies^7,8^ for Bi2223/Ag tapes and melt-textured Dy123 bulks. However, the use of the aforementioned ansatz which can be avoided in the numerical solution of the PDES, without any resulting difference, is insufficient when the aim is to reproduce the experimental results for stacks of 2G-HTS tapes. Then, in order to explain the lower decay in the magnetization observed for stacks of 2G-HTS tapes^4^, we have considered the following ansatz:

2 $$\int {J}_{z}d{{\rm{\Omega }}}_{i}=0\forall \,{\rm{SC}}\,{\rm{tape}}\,{\rm{of}}\,{\rm{cross}}\,{\rm{section}}\,{\rm{area}}\,{{\rm{\Omega }}}_{i}.$$

Equation 2 implies that each one of the SC tapes acts as an individual layer, it being electrically “*unconnected*” to the others (Fig. 2(d)). The use of this ansatz together with Ampère’s law (${\partial }_{x}{H}_{y}-{\partial }_{y}{H}_{x}={J}_{z}$) allows us to see in a straightforward way, how the aspect ratio of each one of the SC tapes plays a primordial role in determining the magnetization decay of the overall stack of tapes. Applying Ampère’s law to the SC domains, produces an equation which is identical to the fundamental Green’s theorem. Thus, the total magnetic flux trapped by each SC layer is defined by the sum of all the “mesoscopic” circulations of the vector field **H** at the positions of the local magnetization currents, ${r}_{i}\in {{\rm{\Omega }}}_{i}$, and is equal to the “macroscopic” circulation of magnetic field around the outer boundary of the SC layer, i.e.,

3 $${\iint }_{{{\rm{\Omega }}}_{i}}(\frac{\partial {H}_{y}}{\partial x}-\frac{\partial {H}_{x}}{\partial y})dxdy={\oint }_{{C}_{i}}({H}_{x}dx+{H}_{y}dy),$$

where *C* is a counterclockwise oriented simple closed curve enclosing the SC domain (see Fig. 2). Then, it is clear that due to the high aspect ratio of the SC tape ($w\parallel x\gg h\parallel y$), any average variation in the total trapped magnetic field in the y-direction and along the *h*-*circulation-paths* is compensated by a much smaller change of the field component *Hx* along the width paths of the SC specimen. Thus, under this ansatz, and for very thin films with aspect ratio comparable to one of 2G-HTS tapes, a significant reduction of the magnetization decay can be envisaged for the crossed and rotating field experiments, although its actual dependence with the relative orientation of the transverse magnetic field needs to be numerically solved. Nevertheless, under the same scheme of the Green’s theorem it is still possible to predict that for rotating field experiments a faster demagnetization process must be observed, it due to the occurrence of a magnetic field in anti-parallel direction to the initial magnetization which reduces the total magnetic field along the h-circulation paths, and concomitantly implies the occurrence of a greater component of magnetic field parallel to the *w*-path of the sample (along the width). Thus, whether our theoretical ansatz is valid, a much lower magnetization decay should be obtained when stacks of 2G-HTS tapes are considered rather than SC bulks, and regardless if the external magnetic field is under the crossed or rotating field configuration, as it is demonstrated in the following section, where we prove by numerical and experimental methods the correctness of our statements.

### Numerical and Experimental Validation

In order to prove our previous statements, in Fig. 2, we show how the local distribution of profiles of current density in a 2G-HTS stack of “interconnected” or “unconnected” tapes, is affected by a transverse magnetic field in the crossed and rotating field configurations. It is worth noting that, when the height and width of the specimen are of similar magnitudes (SC bulks or SC thick films), a strong deformation of the flux front profile (initially vertical), can be obtained after just one cycle of any transverse magnetic field of magnitude comparable to the full-penetration field predicted by Brandt^22^, ${H}_{p\parallel c}\approx {J}_{c}^{\ast }({h}^{\ast }/\pi ){\rm{l}}{\rm{n}}\mathrm{(2}w/{h}^{\ast })$ (Fig. 2(b)). However, when a stack of 2G-HTS tapes is considered, two different results can be obtained as predicted above. For the “interconnected” ansatz, i.e., under the constraint ${\sum }_{{{\rm{\Omega }}}_{i}}^{{{\rm{\Omega }}}_{n}}\int {J}_{z}d{{\rm{\Omega }}}_{i}=0\forall \,i=1,2,\ldots ,n$ SC tapes, the length of the *h-circulating* paths on Eq. 3 is n-times larger than the thickness of a sole SC tape, and therefore the top and bottom layers of the stack become more prone to be demagnetized than the ones under the *unconnected*-ansatz. Quantitatively, after one cycle of the crossed field only about 1% of the initial magnetization is lost for “unconnected” tapes, and up to a 6% can be lost for the system of “interconnected” tapes. On the other hand, for the rotating field experiment, a small band of screening currents on the outermost sides of the SC tapes appears in order to compensate the change in the field density along the y–direction, whilst the integrity of the Green’s theorem is preserved. In this case, we have observed that only about 2% of the initial magnetization is lost for the “unconnected” tapes, compared with about 11% for “interconnected” tapes. In fact, after 100 cycles either of crossed or rotating magnetic field, and under the ansatz of “unconnected” tapes, the sample demagnetization does not exceed 12%, which is in good agreement with our previous experimental evidences for the crossed field effect on 2G-HTS tapes^4^.

**Figure 3:**
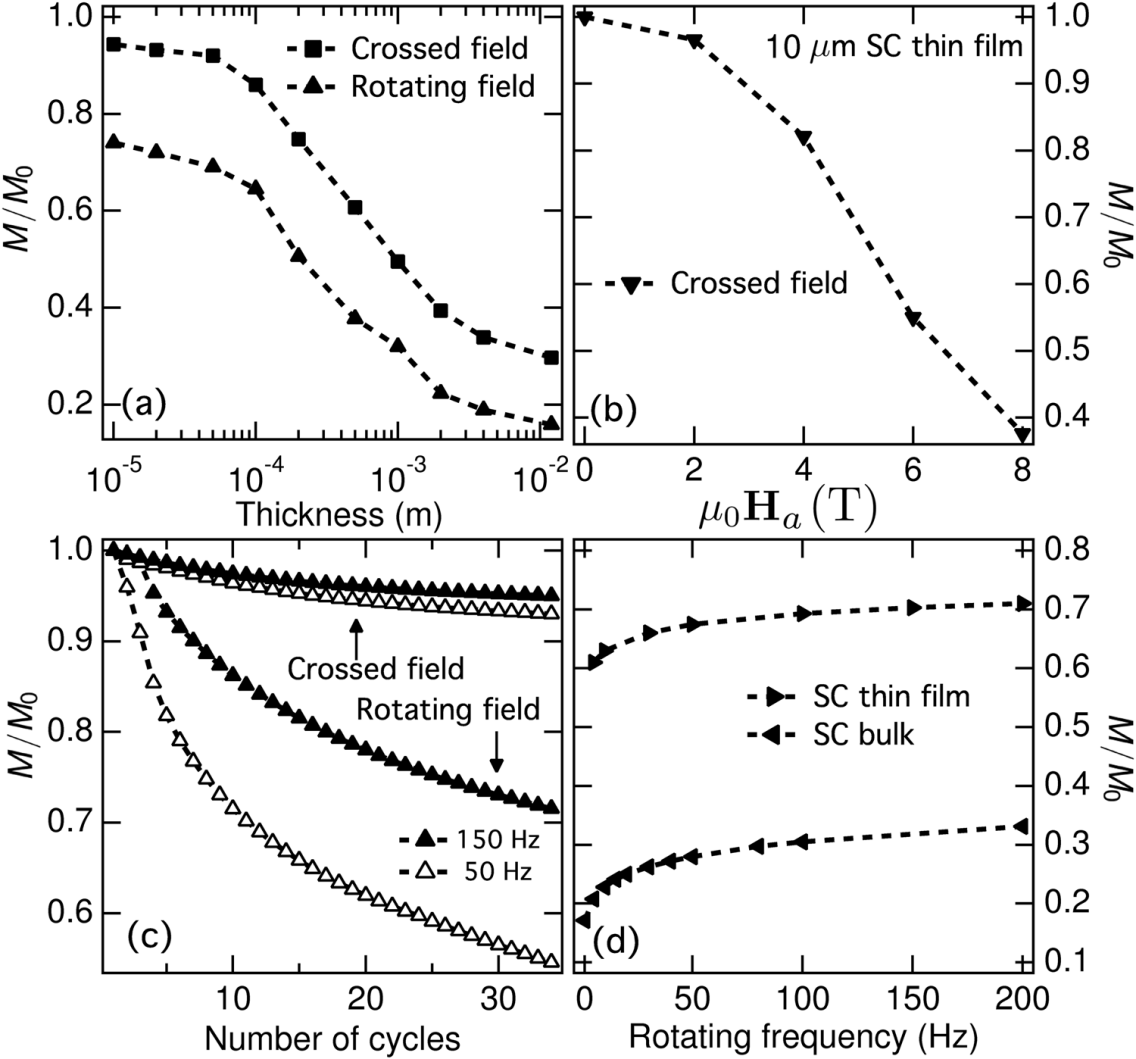
(**a**) Demagnetization rate as a function of the thickness of a fully penetrated SC specimen of 12 mm width, after only one cycle of the crossed- or rotating- field depicted in the subplot (**a**) of Fig. 1, with *H*_a_ = 2 T. (**b**) Analogously, the characteristic dependence with the external magnetic field amplitude is shown for the 10 *μ*m SC sample (thin film), also after one cycle of the crossed field experiment. (**c**) Likewise, the magnetization dependence of the 10 *μ*m SC sample as a function of the number of cycles for the crossed field (top two curves) and rotating field (bottom two curves) are shown for two different frequencies, 50 Hz (open symbols), and 150 Hz (solid symbols). (**d**) Finally, the frequency dependent characteristics at *t* = 0.25t = 0.25 s on the magnetization of the 10 *μ*m SC thin film, is being compared with a SC bulk of aspect ratio (*w*/*h*) = 3, but for the sake of generality, the SC bulk is assumed to have an identical initial magnetization to the one attained by the SC thin film. In this sense, it is to be noticed that all results in this figure are presented in dimensionless units *M*/*M*_0_, with ${M}_{0}=\mathrm{(1/4)}{J}_{c}h{w}^{2}$ the analytical solution for the saturated magnetic moment of a SC sample with longitudinal symmetry^22^.

Before revealing our experimental findings for the rotating field configuration, it is useful to discuss as well the implications of our findings on single thin films of about 10 *μ*m, being this a common thickness for Bi2223 tapes^7^. In Fig. 3(a) we show how, for fully premagnetized SC specimens, thinner than 100 *μ*m (12 mm width), subjected to one cycle of an AC transverse external magnetic field as intense as 2 T, the predicted magnetization decay for the crossed field experiment is lower than 5% of its initial magnetization (*M*_0_), and lower than 40% for the rotating field configuration. *M*_0_ is the Brandt’s analytical solution for the magnetic moment saturation of the SC specimen under a transverse applied magnetic field^22^, which is equivalent to say that the sample considered is initially fully penetrated by a symmetric distribution of profiles of current density *J*_*i*_ = ±*J*_*c*_.

The magnetization decay rapidly increases with thickness reaching a drop of more than 50% for samples thicker than 1 cm. It is worth noting that in order to achieve the same magnetization decay in a 10 *μ*m film, a very large applied magnetic field (*B*_*a*_ > 6 T) would be required under the crossed field configuration (Fig. 3(b)). However, when a rotating magnetic field is considered, the initial magnetization of the SC specimen can be strongly reduced after just a few cycles of an external field of amplitude ${B}_{p}=0.5{\mu }_{0}{J}_{c}w\simeq 140\,mT$ (Fig. 3(c)), although it is still much smaller than the one expected for a SC bulk with identical *M*_0_ (Fig. 3(d)). Thus, although a single thin SC film of about 10 *μ*m thickness shows a superior performance than the one expected for a SC bulk, a relatively steady drop of the initial magnetization (per cycle) is still obtained under an applied rotating magnetic field, directly highlighting the importance of considering stacks of unconnected 2G-HTS tapes (~1 *μ*m each) for the use of superconducting rotary machines.

**Figure 4:**
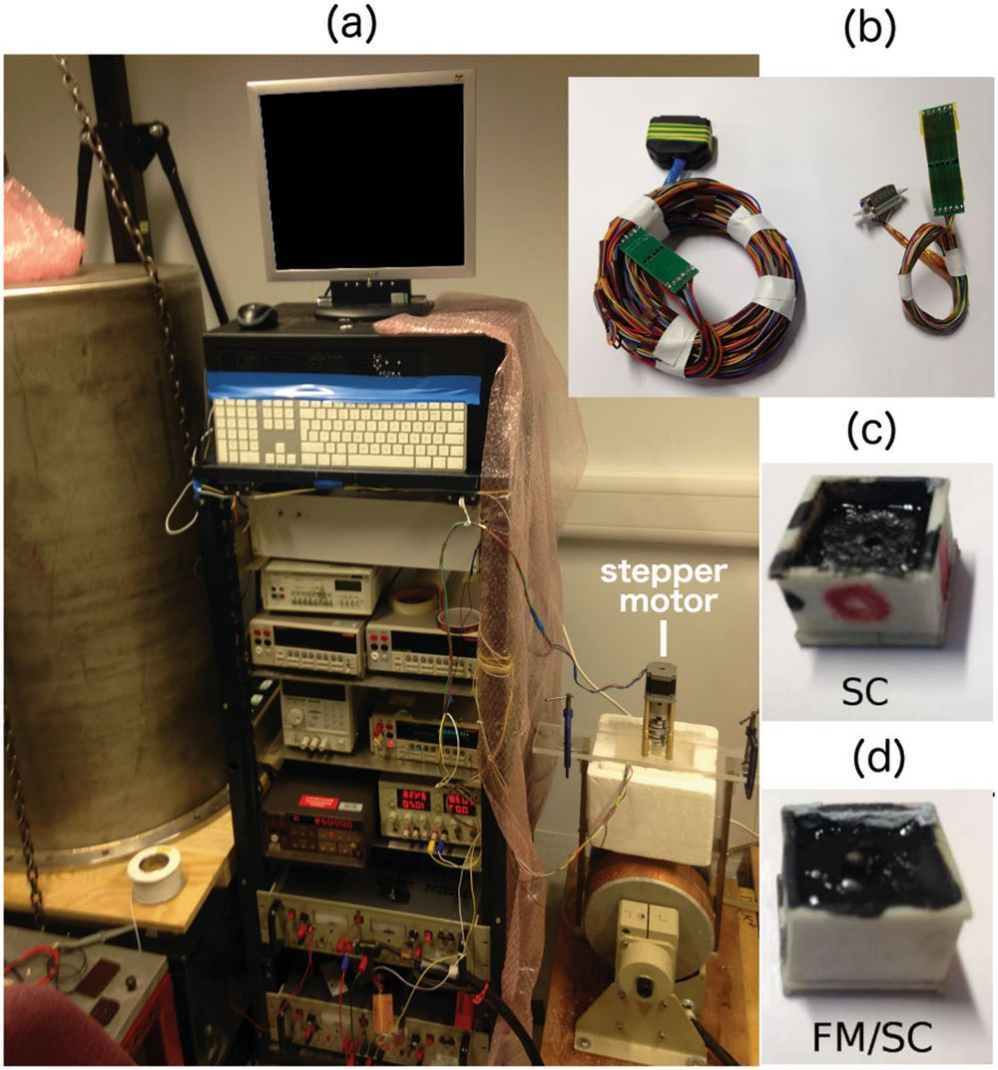
(**a**) Assembled experimental rig for the measurement of the magnetization decay in the rotating and crossed field experiments. (**b**) Set of hall probe boards used for the rotating (left) and crossed (right) field experiments. (**c**) Holder containing the 12 mm SC square stack made of sixteen layers of 2G-HTS tapes. (**d**) Same as above but considering the FM/SC metastructure.

From the experimental perspective, we have validated our theoretical predictions for the stack of 2G-HTS tapes in an experimental rig similar to the one used for crossed field experiments^4^. The main improvement to the original experimental rig has been the adding of a controllable stepper motor at the top of the rod leading the sample holder (Fig. 4), what allows perfoming demagnetization experiments in the rotating field configuration. One of the main issues for conducting rotating field experiments is the difficulty for acquiring data from a printed circuit board of Hall probes that rotates together with the magnetized sample. Therefore, it is necessary to use long enough signal wires to allow the steady control of the system in a low-speed synchronous rotation, without adding excessive load to the motor shaft due to the mechanical stress added by the co-winding of the signal wires. In our case, the experimental rig has been designed for allowing the measurement of 100 rotating cycles under the aforementioned conditions. The samples measured consist of a 12 mm square stack made of sixteen layers of 2G-HTS tapes fabricated by SuperPower Inc.^17^, replicating the fundamental experimental conditions of the crossed field experiments^4^. Additionally, for both the crossed and rotating magnetic field experiments, a second case has been considered by means the interlayering of soft ferromagnetic layers (FM) within the stack of SC tapes (Fig. 1, Case 2), and which we called the SC/FM sample or metastructure. In particular, we have used high purity thin films of soft iron (~99.85%) with a relative magnetic permeability of ~1000 and 20 *μ*m thickness, according to the data-sheet provided by the supplier^23^.

**Figure 5:**
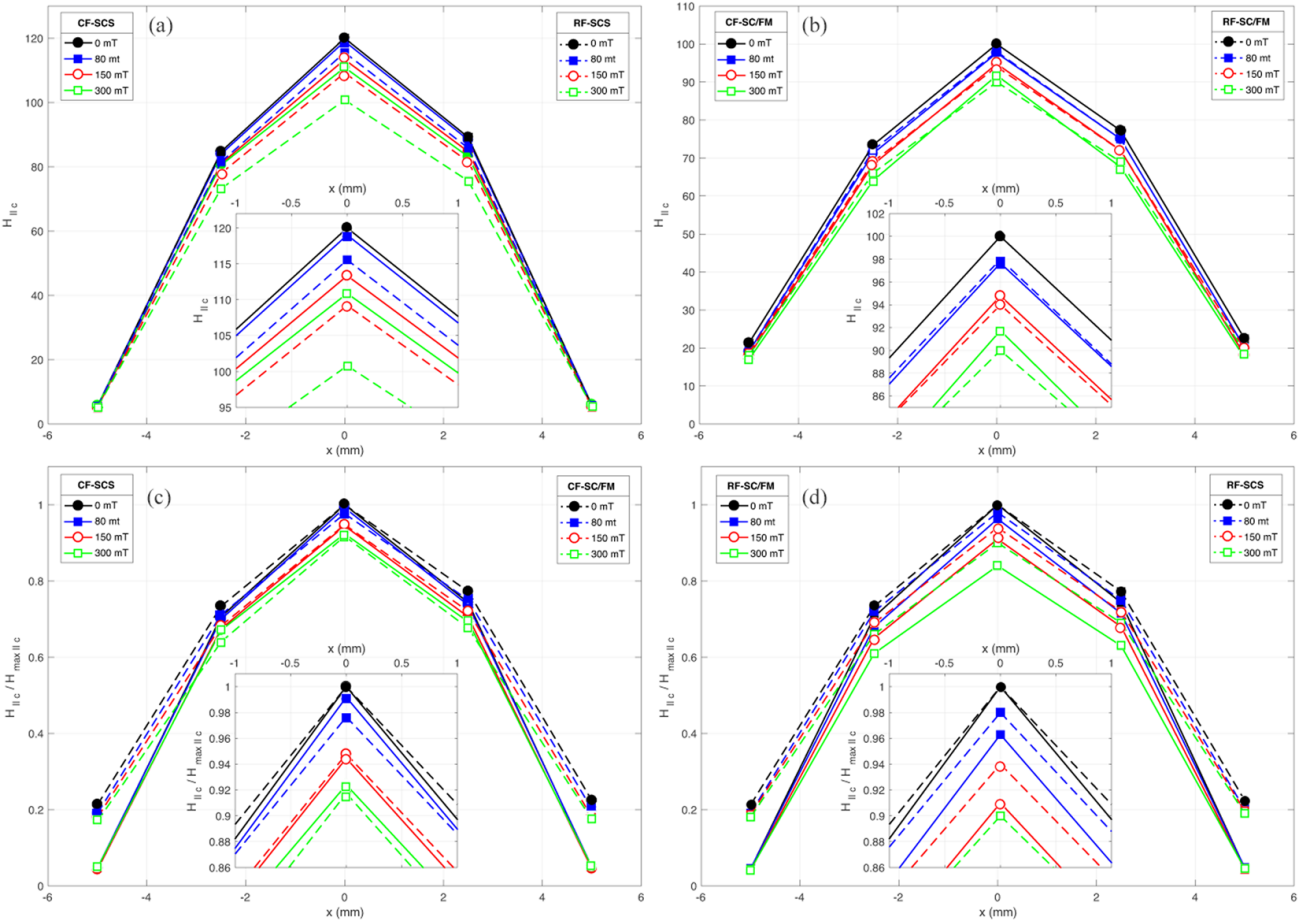
Experimental patterns of intensity of the trapped magnetic field by a 12 mm square stack of sixteen layers of 2G-HTS tapes (SCS) and its analogous SC/FM metastructure illustrated in the inset of Fig. 1, both under the Crossed-Field (CF) and Rotating-Field (RF) configurations. In pane (**a**) the experimental results for the CF-SC (solid lines) and RF-SC (dashed lines) systems are presented. Analogously, the results obtained for the SC/FM sample are displayed in pane (**b**). Then, in panes (**c**) and (**d**) a direct comparison between the SC and SC/FM samples for the CF and RF configurations in normalized units is presented. The insets at each pane shows a zoom of the respective peaks of trapped field at the center of the sample. In all plots, the amplitude of the applied magnetic field for the CF and RF configurations corresponds from top to bottom to i) 0 mT (black lines), i.e., before applying the transverse or rotating magnetic field to the initially magnetized SCS or SC/FM samples, and after 100 cycles of the external magnetic excitation of amplitudes ii) 80 mT (blue lines), iii) 150 mT (red lines), and iv) 300 mT (green lines).

In Fig. 5 we show our experimental results for the intensity of trapped magnetic field before and after applying 100 cycles of the external magnetic excitation within the crossed-field (CF) and rotating-field (RF) configurations, both at the different positions where the main array of Hall probes has been placed^4^. The results are presented for the two different samples introduced above, say the SCS which defines the conventional stack of 2G-HTS tapes, and the SC/FM metastructure. Before considering the CF and RF scenarios, the samples are premagnetized by a 60 seconds field cooling procedure with an external magnetic field of 800 mT, it applied parallel to the c-axis of the 2G-HTS tapes. After the removal of this external field and before applying the new magnetic field for the CF and RF experiments, a time interval of 600 seconds has been allowed for the magnetic relaxation of both samples. Thus, by comparing the profiles of trapped magnetic field for the SCS (Fig. 5(a)) and the SC/FM (Fig. 5(b)), it is to be noticed that by adding FM interlayers to the SCS, the resulting SC/FM metastructure requires a greater applied magnetic field in order to trap the same amount of field trapped by the SCS at the center of its top-surface (*x* = 0 mm). Nevertheless, a more acute peak of trapped field is observed for the SCS sample as it is shown in Fig. 5(c,d), where the experimental patterns for the CF and RF are show in renormalized units for each of the SCS and SC/FM samples. Being more precise, the maximum trapped magnetic field along the c-axis of the SCS is ${\mu }_{0}{H}_{max\parallel c}\approx 120$ mT, i.e., ~20% more than the maximum trapped magnetic field for the SC/FM, ${\mu }_{0}{H}_{max\parallel c}\approx 100$ mT. However, the space variation of trapped magnetic field towards the edges of the SC/FM metastructure is smaller than the pattern observed for the SCS sample, with a trapped magnetic field of ~20% mT at *x* = ±5 mm, it compared with the ~6mT trapped by the SCS at the same positions.

A striking characteristic of the SC/FM metastructure is that for the same intensity of applied magnetic field, the difference in the magnetization decay between the CF and RF experiments is almost negligible (Fig. 5(b)), contrary to what happen with the SCS sample (Fig. 5(a)) where a RF leads to a more noticeable drop in the peak of trapped magnetic field. Thus, although compared with a superconducting bulk of equivalent dimensions^4^, the SCS sample under RF conditions still shows a remarkably strong reduction of the magnetization decay even after 100 cycles of an external magnetic field of 300 mT amplitude (~20%), it is ~10% greater that the magnetization decay observed for the CF configuration with the same intensity and number of cycles of applied field. It is worth reminding that for the case of a SC bulk, the remnant magnetization signal after just a couple of cycles of the CF is nearly zero^14^ for much lower amplitudes of magnetic field as the one considered here. However, with the use of a SC/FM metastructure, not only does the undesired decrease in the trapped magnetic field reduce by half within CF conditions, but also it does not show significant variations under rotating magnetic fields. This experimental finding is of utter relevance as it may allow a more extended use of the SC/FM metastructures for high field rotating machines rather than the simple use of SCS samples which have resulted to be more prone to demagnetization effects by rotating fields (Fig. 5(d)). In fact, in quantitative terms, an average difference of just ~2% has been observed between the CF and RF experiments with the SC/FM metastructure as it can be observed in Fig. 5(b).

**Figure 6:**
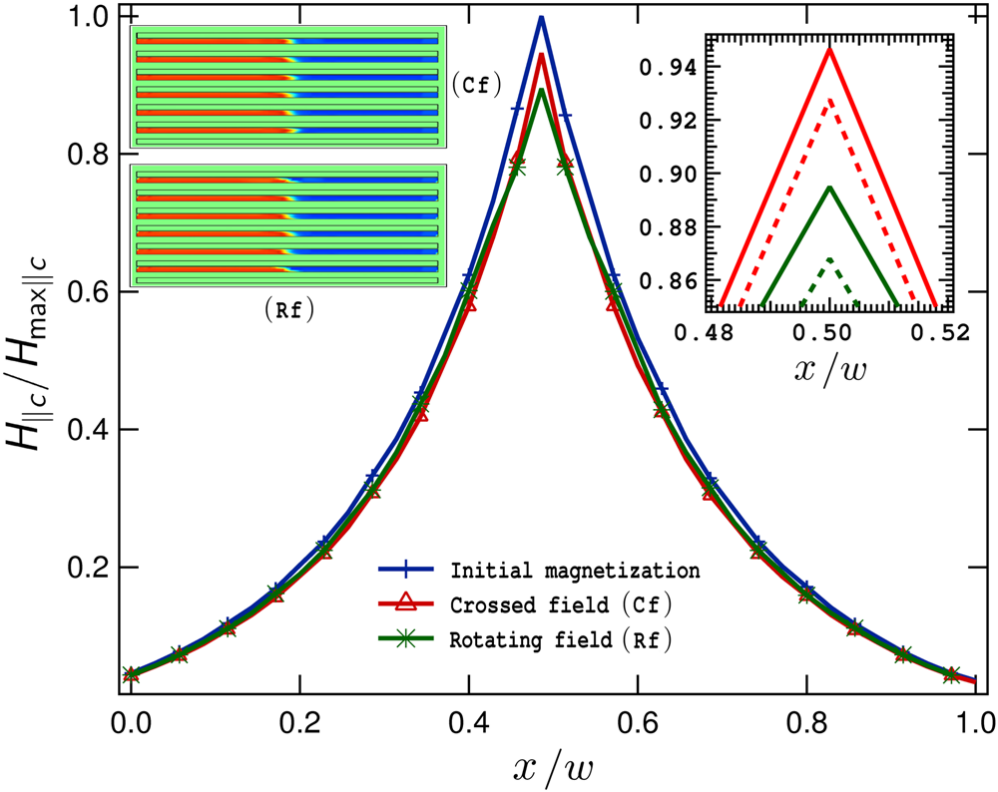
Renormalized intensity of the trapped magnetic field (${H}_{\parallel c}/{H}_{{\rm{\max }}\parallel c}$) over the upper surface of the SC/FM metastructure illustrated at the left side inset (not to scale), before and after 50 cycles of the crossed (top) or rotating (bottom) magnetic field experiment, respectively. At the right inset, the drop of the peak after 100 cycles (solid lines) is shown by comparison.

Aiming to provide a further validation to our experimental and theoretical findings for the RF configuration and how these extend to the case of SC/FM metastructures, we have additionally performed a numerical computation of the trapped magnetic field by a generic SC/FM metastructure subjected to RF conditions. Thus, in order to reduce the computing time required for solving up to 100 cycles of the RF in samples where the aspect ratio plays a significant physical and computational role but yet being able to demonstrate the generality of our statements, we have considered a stack of six 2G-HTS tapes with interlayered films of a soft-ferromagnetic material of the same thickness than the used in our experiments (20 *μ*m), but with the magnetic permeability analogous to the one used for the second case of study in Fig. 1. The reason of this choice, *μ*_*r*_ = 100, is justified by the observations of other groups^24,25^, where it has been established than the use of a soft FM layer with a relative magnetic permeability of *μ*_*r*_ = 100–200, is sufficient for an effective screening of a transverse magnetic field with no apparent difference in the limit *μ*_*r*_ = ∞. Hence, for a fully premagnetized SC/FM metastructure, the FM layers act to prevent any change in the direction of the magnetic field over the surface of the SC layers. Then, for the CF and RF experiments, this fact implies that no matter what the direction of the transverse magnetic field is, the macroscopic circulation of the vector *H* remains unaltered accordingly to Eq. 3, and consequently, very small demagnetization effects can be expected in the actual measurements as it has been experimentally demonstrated in Fig. 5, and as it has been theoretically proven in Fig. 6. Therein, it can be observed than an exceptional conservation of the initial profile of magnetic field across the width of the sample can be predicted for even up to 100 cycles of the RF, in good agreement with our experimental measurements for a SC/FM metastructure more than twice thicker than the one considered in our numerical approach.

### Conclusions

In this paper, we have demonstrated that the striking finding of a very low magnetization decay recently observed^4^ for stacks of 2G-HTS tapes under the crossed field configuration, can be explained by using the conventional framework of the **H**-Formulation^12,14^ without any discrepancy with reported theoretical models for Bi2212/Ag tapes, SC bulks, and other structures of similar size^7,8^, by showing that the set of 2G-HTS tapes physically behaves as a system of electrically “unconnected” layers (Eq. 2), with the overall aspect ratio of the specimen being large enough to avoid the deformation of the profiles of current along the circulation path defined by the Green’s theorem. Then, in order to provide a further validation of our theoretical ansatz which by physical consistence must remain valid regardless the direction of the transverse magnetic field, we have presented new experimental findings together with a comprehensive numerical study for the so-called configurations of rotating field (RF) and crossed field (CF) experiments, demonstrating the validness of our physical ansatz for the proof of the low magnetization decay observed in stacks of 2G-HTS tapes, after even 100 cycles of the external magnetic excitation.

For the premagnetization stage, we have determined that the optimal band for sizing the height (h*) of a stack of 2 G HTS tapes of width *w* is $1\le {h}^{\ast }/w\le 2$, with a rapid decay of the trapped magnetic flux for samples with aspect ratio h*/*w* < 1, and with tendency to saturation for h*/*w* > 2. On the other hand, as it is shown in Fig. 1 (Case 2), by interlayering thin films of a soft ferromagnetic material it is possible to substantially increase the amount of trapped magnetic field for samples of aspect ratio greater than h*/*w* = 1 (~22% increment), saturating about h*/*w* = 8 (~70% increment). Then, for the CF and RF conditions, we have confirmed our theoretical predictions for the low decay in the magnetization of stacks of 2G-HTS tapes by performing the actual measurement of the drop of trapped magnetic field across the width of the samples with and without FM layers. Remarkably, we have found that for the same intensity of transverse magnetic field applied to the SC/FM sample, the difference between the magnetization decays obtained by the CF and RF configurations is nearly zero, and these decays are reduced by half when they are compared with the conventional stack of 2G-HTS tapes (SCS). In fact, we have experimentally found that after 100 cycles of an external magnetic field of 300 mT in the CF or RF configurations, the drop in the peak of trapped magnetic field for the SC/FM metastructure is just of 10%, in contrast to a 20% drop observed for the SCS sample in RF conditions. Our results for the SC/FM metastructure have been also validated by numerical methods, adding further insights on the multiple benefits offered by the use of SC/FM metastructures for multiple engineering applications^26–28^.
